# Protocol for the development of guidance for collaborator and partner engagement in health care evidence syntheses

**DOI:** 10.1186/s13643-023-02279-1

**Published:** 2023-08-02

**Authors:** Peter Tugwell, Vivian Welch, Olivia Magwood, Alex Todhunter-Brown, Elie A. Akl, Thomas W. Concannon, Joanne Khabsa, Richard Morley, Holger Schunemann, Lyubov Lytvyn, Arnav Agarwal, Alba Antequera, Marc T. Avey, Pauline Campbell, Christine Chang, Stephanie Chang, Leonila Dans, Omar Dewidar, Davina Ghersi, Ian D. Graham, Glen Hazlewood, Jennifer Hilgart, Tanya Horsley, Denny John, Janet Jull, Lara J. Maxwell, Chris McCutcheon, Zachary Munn, Francesco Nonino, Jordi Pardo Pardo, Roses Parker, Kevin Pottie, Gabriel Rada, Alison Riddle, Anneliese Synnot, Elizabeth Tanjong Ghogomu, Eve Tomlinson, Karine Toupin-April, Jennifer Petkovic

**Affiliations:** 1 Department of Medicine, Faculty of Medicine, University of Ottawa, Ottawa, Canada; 2 Ottawa Hospital Research Institute, Clinical Epidemiology Program, Ottawa, Canada; 3 School of Epidemiology and Public Health, Faculty of Medicine, University of Ottawa, Ottawa, Canada; 4 WHO Collaborating Centre for Knowledge Translation and Health Technology Assessment in Health Equity, Bruyère Research Institute, Ottawa, Canada; 5 Bruyere Research Institute, Ottawa, Canada; 6 Interdisciplinary School of Health Sciences, University of Ottawa, Ottawa, Canada; 7 Nursing Midwifery and Allied Health Professions (NMAHP) Research Unit, Glasgow Caledonian University, Glasgow, UK; 8 Department of Internal Medicine, American University of Beirut, Beirut, Lebanon; 9 Department of Health Research Methods, Evidence, and Impact (HEI), McMaster University, Hamilton, ON Canada; 10 The RAND Corporation and Tufts University School of Medicine, Boston, MA USA; 11 Clinical Research Institute, American University of Beirut Medical Center, Beirut, Lebanon; 12 Cochrane, London, UK; 13 Department of Health Research Methods, Evidence, and Impact, Michael G DeGroote Cochrane Canada Centre, Cochrane Canada and McMaster GRADE Centre, McMaster University, Hamilton, ON Canada; 14 Department of Medicine, McMaster University, Hamilton, ON Canada; 15 Department of Biomedical Sciences, Humanitas University, Milan, Italy; 16 Cochrane Canada, Hamilton, ON Canada; 17 McMaster University, Hamilton, Canada; 18 Division of General Internal Medicine, Department of Medicine, McMaster University, Hamilton, ON Canada; 19 International Health Department, ISGlobal, Hospital Clínic-Universitat de Barcelona, Barcelona, Spain; 20 Canadian Council On Animal Care, Ottawa, Canada; 21 Agency for Healthcare Research and Quality, Rockville, MD USA; 22 Annals of Internal Medicine, Philadelphia, PA USA; 23 Department of Clinical Epidemiology, University of the Philippines-Manila, Manila, Philippines; 24 Research Translation, National Health and Medical Research Council, Canberra, Australia; 25 Sydney Medical School, The University of Sydney, Sydney, Australia; 26 Centre for Implementation Research, Ottawa Hospital Research Institute, Ottawa, Canada; 27 Cumming School of Medicine, University of Calgary, Calgary, Canada; 28 Cochrane Central Executive Team, London, UK; 29 Royal College of Physicians and Surgeons of Canada, Ottawa, Canada; 30 PharmaQuant, Kolkata, India; 31 Center for Public Health Research (CPHR), Kolkata, India; 32 School of Rehabilitation Therapy, Queen’s University, Kingston, ON Canada; 33 Faculty of Medicine, University of Ottawa, Ottawa, Canada; 34 Faculty of Health and Medical Sciences, JBI, University of Adelaide, Adelaide, South Australia Australia; 35 Unit of Epidemiology and Statistics, IRCCS Istituto delle Scienze Neurologiche di Bologna, Bologna, Italy; 36 Ottawa Hospital Research Institute, Ottawa Methods Centre, Ottawa, Canada; 37 Cochrane Pain Palliative and Supportive Care, Oxford University Hospitals Trust, Oxford, England; 38 Departments of Family Medicine and Epidemiology and Biostatistics, Western University, London, ON Canada; 39 Department of Family Medicine, University of Ottawa, Ottawa, Canada; 40 Epistemonikos Foundation, Santiago, Chile; 41 UC Evidence Centre and Department of Internal Medicine, Pontificia Universidad Católica de Chile, Santiago, Chile; 42 School of Public Health and Preventive Medicine, Monash University, Level 4, 553 St Kilda Road, Melbourne Victoria, 3004 Australia; 43 Centre for Health Communication and Participation, School of Public Health and Psychological Sciences, La Trobe University, Plenty Rd, Bundoora, VIC 3086 Australia; 44 Bristol Medical School, Population Health Sciences, University of Bristol, Bristol, UK; 45 School of Rehabilitation Sciences, Faculty of Health Sciences, University of Ottawa, Ottawa, Canada; 46 Department of Pediatrics, Faculty of Medicine, University of Ottawa, Ottawa, Canada; 47 Children’s Hospital of Eastern Ontario Research Institute, Ottawa, Canada; 48 Institut du Savoir Montfort, Ottawa, Canada

**Keywords:** Systematic reviews, Stakeholder, Engagement, Evidence synthesis, Coproduction

## Abstract

**Background:**

Involving collaborators and partners in research may increase relevance and uptake, while reducing health and social inequities. Collaborators and partners include people and groups interested in health research: health care providers, patients and caregivers, payers of health research, payers of health services, publishers, policymakers, researchers, product makers, program managers, and the public. Evidence syntheses inform decisions about health care services, treatments, and practice, which ultimately affect health outcomes.

Our objectives are to:

A. Identify, map, and synthesize qualitative and quantitative findings related to engagement in evidence syntheses

B. Explore how engagement in evidence synthesis promotes health equity

C. Develop equity-oriented guidance on methods for conducting, evaluating, and reporting engagement in evidence syntheses

**Methods:**

Our diverse, international team will develop guidance for engagement with collaborators and partners throughout multiple sequential steps using an integrated knowledge translation approach:

1. Reviews. We will co-produce 1 scoping review, 3 systematic reviews and 1 evidence map focusing on (a) methods, (b) barriers and facilitators, (c) conflict of interest considerations, (d) impacts, and (e) equity considerations of engagement in evidence synthesis.

2. Methods study, interviews, and survey. We will contextualise the findings of step 1 by assessing a sample of evidence syntheses reporting on engagement with collaborators and partners and through conducting interviews with collaborators and partners who have been involved in producing evidence syntheses. We will use these findings to develop draft guidance checklists and will assess agreement with each item through an international survey.

3. Consensus. The guidance checklists will be co-produced and finalised at a consensus meeting with collaborators and partners.

4. Dissemination. We will develop a dissemination plan with our collaborators and partners and work collaboratively to improve adoption of our guidance by key organizations.

**Conclusion:**

Our international team will develop guidance for collaborator and partner engagement in health care evidence syntheses. Incorporating partnership values and expectations may result in better uptake, potentially reducing health inequities.

**Supplementary Information:**

The online version contains supplementary material available at 10.1186/s13643-023-02279-1.

## Summary

An evidence synthesis, sometimes called a systematic review, is a method which identifies, brings together, and analyses all the research studies which address a specific question about healthcare. They are often used to provide evidence for healthcare decisions, such as the therapies that will be recommended by a physician, or for public health. Including people and groups who have an interest in these decisions, such as, patients, healthcare providers, those who pay for health services, those who manage health programs, and others, can help make sure that the right questions are asked, and the right information is assessed. The best way to engage with these all these groups has not been identified. The goal of this project is to develop guidance for engaging with multiple groups or ‘collaborators and partners’ in each step of the evidence synthesis process. To prepare this guidance, we will identify and synthesize the available information on collaborator and partner engagement in evidence synthesis, conduct interviews and a survey and hold international meetings to develop and finalise draft checklists for collaborator and partner engagement in evidence synthesis.

## Background

Evidence syntheses are used to inform the development of clinical practice, health systems, and public health guidelines [1]. While the rate of evidence synthesis production is steadily increasing there are serious questions about whether they are useful, meaningful, or accessible [2–4]. Given these challenges, as well as the associated costs of conducting evidence synthesis, maximizing their impact is important [5]. Engaging collaborators and partners in synthesising evidence that is meaningful to them improves equity and accessibility and the overall quality of the synthesis [6].

There is international recognition that the engagement of collaborators and partners in health care research is important for improving its impact. Meaningful engagement benefits the usefulness, relevance, quality, buy-in, uptake, and impact of research [7–9]. For example, engaging patients and the public can increase researchers’ understanding of the issues, appropriateness of the research, and interpretation of findings [8, 10]. High-quality research, co-produced with all key collaborators and partners, is fundamental to supporting the reduction of research waste and promoting equity [11]. Engagement can identify evidence gaps and refine scope, address barriers to the uptake of evidence, increase dissemination and application of findings, and thus help formulate recommendations for research [11–14].

There has been an increase in research teams undertaking collaborator and partner engagement in evidence synthesis as well as greater expectations from funders, but reporting of engagement is poor and there are evaluations or guidance about how to effectively engage different groups [15–18]. In parallel, there is a growing body of evidence providing guidance for collaborator and partner engagement in research and health care guidelines [16, 19–21]. Despite this, existing evidence predominantly focuses on patient and public involvement in primary research with a notable lack of tailored guidance for engaging other groups in evidence synthesis development [7, 8, 22, 23].

However, the most effective methods for engaging different collaborators and partners in evidence synthesis, identifying the barriers or facilitators for engagement, or reporting how collaborators and partners were engaged have not been identified. The Multi-Stakeholder Engagement (MuSE) Consortium has recently addressed these issues in relation to health guidelines [16]. Lessons learned from the guidelines project will inform this work as applicable. The current project aims to apply the lessons learned from that project to the conduct of evidence syntheses. We will develop guidance for collaborator and partner engagement in health care evidence syntheses to facilitate the production of relevant and useful evidence syntheses.

## Study aim

Our overarching goals are to synthesize evidence relating to collaborator and partner engagement in health care evidence syntheses and to explore perspectives on how engagement in evidence syntheses can promote health equity. Our specific objectives are to:

A) Identify, map, and synthesize qualitative and quantitative findings related to collaborator and partner engagement in evidence syntheses
B) Explore perspectives on how collaborator and partner engagement in evidence syntheses promotes health equity
C) Develop equity-oriented guidance on methods for collaborator and patient engagement in evidence synthesis
D) Develop guidance on methods for evaluating collaborator and partner engagement in evidence syntheses
E) Develop a guideline for reporting collaborator and partner engagement in evidence syntheses (Preferred Reporting Items for Systematic Reviews and Meta-Analyses (PRISMA) extension).

### Definitions of key terms

Definitions of terms in this field often lack consistency and differ internationally [19]. For this project, we use the following definitions:

#### Health care evidence syntheses

Synthesize research evidence to address a health care question using rigorous, explicit, and transparent methods. These include scoping reviews, rapid reviews, and quantitative or qualitative systematic reviews [24].

#### Collaborator and partner

Any interested person or group who is responsible for or affected by health- and healthcare-related decisions [25]. We previously used the term 'stakeholder' to describe these groups, however the historical use of the word and its relation to colonialism is problematic. Through discussions with the MuSE Consortium members, we have decided to replace the term with 'collaborators and partners'. For this project we have grouped collaborators and partners into the following 11 categories, which we call the 11 ‘Ps’. These categories are designed to be comprehensive of all interested people or groups; however, we are cognisant that roles and terminology vary internationally, and some collaborators and partners may fit under multiple categories:

- Patients/consumers, caregivers, and patient groups
- Payers/funders of research
- Payers and purchasers of health services (e.g. those who pay for or reimburse health-related interventions, including insurers, individuals with deductibles, others, and those entities responsible for underwriting the cost of care, such as employers and governments)
- Publishers (those involved in the knowledge translation of evidence syntheses, e.g. peer-review editors, scientific publishers, science writers)
- Policy-makers (e.g. governments and professional associations, those involved in the regulatory processes of drugs and health devices)
- Principal investigators (e.g. researchers conducting studies that may or may not be relevant to the review)
- Product makers (e.g. drug, natural products and/or device manufacturers)
- Producers and commissioners of guidelines (e.g. institutions and organizations that commission, develop, or implement guideline development procedures) [26].
- Program managers (e.g. managers/directors/administrators and individuals who plan, lead, oversee, or deliver any program that provides public health, community services, or clinical care (e.g. budgeting, hiring, staffing, organizing, coordinating, reporting). These individuals may be health care providers but are not on the point of care delivering health care related to the program of interest (e.g. overseeing an immunisation program but not delivering vaccinations)
- Providers (individuals and/or organizations providing care, such as nurses, physicians, pharmacists, community-based workers)
- Public (e.g. communities or general members of the population or community, excluding patients, caregivers, and health professionals, living or working with the condition of interest)

#### Conflict of interest

“A conflict of interest exists when a past, current or expected interest creates a significant risk of inappropriately influencing an individual’s judgment, decision, or action when carrying out a specific duty” [26] and may be related to financial, intellectual or other interests. Interest refers to a benefit (e.g. money received from industry) or to an attribute of the individual (e.g. having specific beliefs about religion, evidence-based medicine).

#### Engagement

Refers to the approach to gather input or contribution from collaborators and partners and is multi-directional, resulting in “informed decision-making about the selection, conduct, and use of the research” [27]. Language varies internationally, with other terms including “partnerships”, “involvement”, “consultation”, “co-production”, “co-creation”, and “Patient and Public Involvement (PPI)” [19].

#### Health equity

“The absence of unfair, avoidable or remediable differences among groups of people, whether those groups are defined socially, economically, demographically, or geographically or by other dimensions of inequality” [28]*.* We use the acronym PROGRESS-Plus to identify characteristics which may contribute to health inequities (place of residence (e.g. country), race/ethnicity/culture, occupation, gender or sex and other identities, religion, education, socioeconomic status, social capital, and other characteristics including age or career stage) and to identify groups who may be underrepresented in evidence synthesis [29].

#### The MuSE consortium

The MuSE Consortium is a group of over 160 individuals from 20 countries representing our 11 'Ps' who each have an interest in collaborator and partner engagement in health research, evidence syntheses, and guidelines. This project is complementary to another MuSE project which aims to develop guidance for engagement in health guidelines [16].

## Methods

### Methodological approach

Our methodological approach is adapted from the guidance for developing research reporting guidelines [30] and our previous project to develop guidance for collaborator and partner engagement in health guideline development [16]: identifying the need for guidance; reviewing the literature; generating a list of candidate items for consideration; conducting key informant interviews to refine the candidate items; conducting a survey to assess agreement with candidate items; holding an in-person consensus meeting; and developing, piloting of draft guidance items an publishing and disseminating the guidance [31].

This project uses a multiple mixed methods design. We will triangulate the findings of the evidence syntheses, methodological study, and interviews to develop a set of draft guidance items related to collaborator and partner engagement in evidence synthesis [32]. The series of evidence syntheses will be planned a priori with separate published protocols. However, step 2 may include emergent components to enable us to explore issues identified in the literature [33].

### Positionality statement

The research team includes contributors to the Campbell and Cochrane Collaborations. Our team is comprised of individuals representing our identified collaborator and partner categories (11 Ps) who are all committed to the value of collaborator and partner engagement in evidence synthesis as well as improving the reporting and evaluation of engagement.

### Integrated knowledge translation

**Fig. 1:**
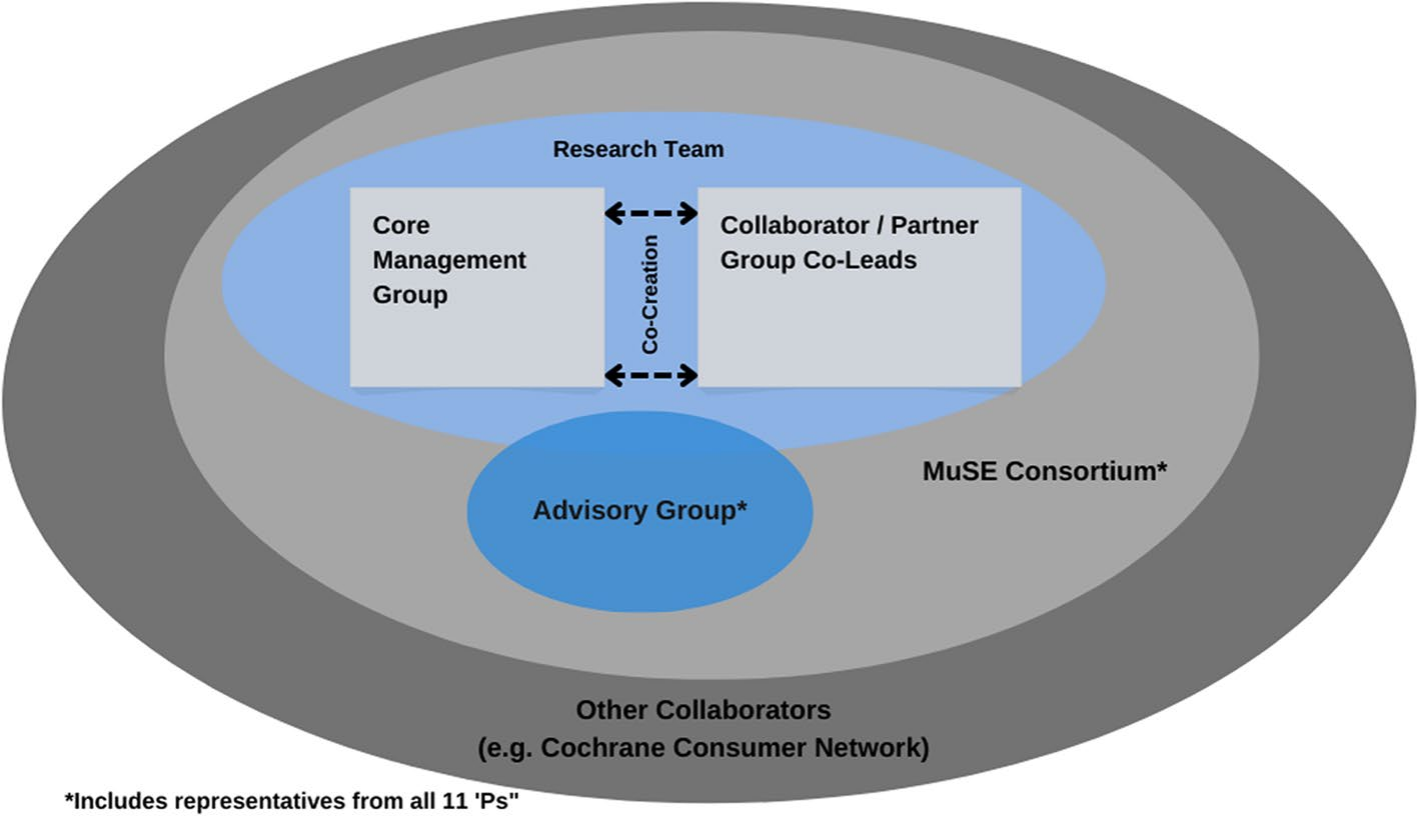
Project team

We have assembled an international core management group which has developed this protocol with our collaborator and partner categories (11 Ps). The core management group will manage the day-to-day aspects of the projects. We are also establishing an international advisory group which will include additional representatives of our 11 Ps. They will be invited to engage with the co-leads of their collaborator and partner category to develop the draft guidance items for their group. We will provide further opportunities for a wide range of relevant collaborators and partners, including other members of the MuSE Consortium and Cochrane Consumers. Please see Fig. 1 and Additional file 1. We will engage with all members of the MuSE Consortium through quarterly newsletters and will invite them to provide feedback throughout the project.

### Project design

**Fig. 2:**
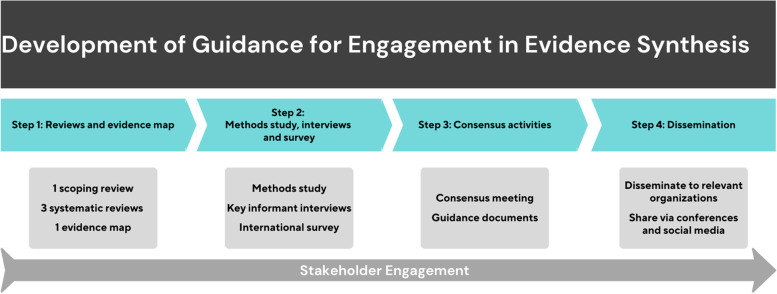
Project plan

This project has four sequential steps that will result in the development of consensus-driven guidance (Fig. 2) [34]. We will first conduct a series of evidence syntheses: one scoping review, three systematic reviews and one evidence map. We will conduct a descriptive assessment of published evidence syntheses which report how collaborators and partners were engaged in the process. We will solicit additional details and contextual information about collaborator and partner engagement through key informant interviews so that we can draft items to be included in the guidance documents. We will seek broad input into these guidance items through an international survey. The results of the survey will be taken to a consensus meeting which will aim to finalise the guidance which will then be widely disseminated. The following sections describe each step of the process.

### Step 1: evidence syntheses

#### Description of the series of evidence syntheses

We will complete four reviews and an evidence map to identify and synthesize qualitative and quantitative findings related to collaborator and partner engagement. The topics of these reviews are:

- Collaborator and partner engagement methods (scoping review and evidence map)
- Barriers and facilitators to collaborator and partner engagement in conduct of evidence synthesis (systematic review)
- Impact of collaborator and partner engagement in evidence synthesis (systematic review)
- Conflicts of interest in collaborator and partner engagement in evidence synthesis (systematic review)
- Equity considerations in collaborator and partner engagement in evidence synthesis (evidence map)

We will follow established methods for high quality evidence syntheses and evidence mapping and publish separate, detailed protocols review will using the Open Science Framework [31, 35–37]. Table 1 describes the outcomes of interest and provides an example of an included study for each of the reviews and the evidence map. Briefly, we will coordinate this series of reviews by conducting one broad comprehensive search for published literature combining concepts of “collaborators" and "partners”, “engagement”, and “evidence synthesis”. We will develop and test comprehensive search strategies in consultation with an experienced information specialist which will include electronic databases as well as an extensive grey literature search. The strategies will be peer reviewed using the PRESS checklist [38]. We will adapt the search of the previous scoping review and search databases including health databases (e.g. MEDLINE and CINAHL) as well as sociological, psychological, and international development databases. We will use the citation chaser tool to identify additional potentially relevant studies [39]. This tool conducts backward and forward citation tracking. We will also search the websites of institutions of agencies that produce evidence syntheses and their methods, such as JBI, and the EPPI-Centre. We will not include papers describing engagement in health technology assessments (HTA). While the methods used for evidence synthesis within HTAs are similar, the process is quite distinct and is often more responsive to policy needs [40].

**Table 1:** Description of each review

Review topic	Type of review	Aim	Phenomena of interest	Outcomes of interest	Studies eligible	Example of eligible study
Methods to engage collaborators and partners	Scoping review	To summarize how collaborators and partners have been involved in evidence synthesis	Methods used for engaging collaborators and partners throughout the evidence synthesis process	Methods of engaging with different collaborators and partners	Papers reporting on methods used for engaging collaborators and partners in evidence synthesis	A review assessing how the public have been involved in systematic reviews, including at an organizational level as well as the which stages of individual reviews [41]
Barriers and facilitators to collaborator and partner engagement	Qualitative evidence synthesis	To synthesize the barriers and facilitators to collaborator and partner engagement in evidence synthesis	Collaborator and partner engagement in evidence synthesis	Barriers and facilitators to collaborator and partner engagement	Papers reporting on barriers and/or facilitators for engaging with collaborators and partners	A survey of patient research partners asking about their participation in the analysis of a qualitative meta-synthesis [42]
Impacts of collaborator and partner engagement	Systematic review	To synthesize the impacts of collaborator and partner engagement on the evidence synthesis process, the product, its uptake and on the collaborators and partners involved	The impacts of collaborator and partner engagement on evidence synthesis	The impact on the evidence synthesis product, on those producing the synthesis, the research process, and wider dissemination	Empirical evaluations (quantitative or qualitative) of the impacts of collaborator and partner engagement in evidence synthesis	Paper describing end-user involvement in a systematic review of ADHD in schools which reflects on the impact of involvement and highlights challenges and benefits experienced [43]
Conflicts of interest	Systematic review	To synthesize the literature on managing conflicts of interest related to collaborator and partner engagement	COI issues for collaborator and partner engagement in evidence synthesis	The type and prevalence of relevant COI by collaborator and partner category, impacts of conflicts on the synthesis process and on collaborator and partner perceptions, and approaches for management of COI	Studies describing issues related to COI in evidence synthesis and methods for their management	A study that assessed the frequency and types of COI reported by authors of systematic review on health policy and systems research [44]
Equity considerations for collaborator and partner engagement in evidence synthesis	Scoping review and evidence map	To identify, summarise, and map the equity considerations included in existing tools, frameworks, models, and checklists for evidence synthesis in partnership with collaborators and partners To map the impact of collaborators and partners engagement in evidence synthesis across equity characteristics (PROGRESS-Plus)	Equity considerations for collaborator and partner engagement in evidence synthesis	Methods (values, principles, guidance) for promoting equitable engagement of collaborators and partners in evidence synthesis; Impacts of collaborator and partner engagement in evidence synthesis across equity characteristics (defined by PROGRESS-Plus)	Papers that report the development of a framework/model and/or evaluation of collaborator and partner engagement in evidence synthesis	A paper describing the methods of involvement young people with lived experience with long-term physical conditions and mental health issues in a complex evidence synthesis [45]

Records will be imported into Covidence (https://www.covidence.org/) and de-duplicated. Pairs of independent reviewers will conduct title and abstract screening, tagging potentially relevant studies for inclusion into any of our reviews. Full text “tagged” papers will be retrieved for each of the individual reviews and will be considered alongside specific eligibility criteria. Data from studies judged as relevant will be extracted by two independent reviewers and the methodological quality of the evidence assessed, as appropriate, for each review.

We will invite input from MuSE members and our advisory group on our planned search strategies. To include additional perspectives, we will invite recommendations for literature to be included in these reviews by reaching out to non-MuSE members to ensure we capture published and unpublished literature using social media and through our mailing lists.

### Step 2: methods study, interviews, and survey

#### Methods study

To expand on the findings of the evidence syntheses conducted in step 1, we will conduct a descriptive assessment of a random sample of published evidence syntheses that have engaged with collaborators and partners. Using the search strategy developed for step 1, we will identify examples of published evidence syntheses that report on engagement with collaborators and partners in their abstract. Two reviewers will independently screen that titles and abstracts of potential evidence syntheses to assess their eligibility. We will include any evidence synthesis that reports on engagement with any of our identified collaborator and partner categories at any stage of the evidence synthesis process.

We will export the records to Microsoft Excel and sort them randomly using the built-in random number generator. We will review the full texts of the evidence syntheses until we have reached our target sample size of 100 evidence syntheses that report on collaborator and partner engagement.

We will develop and pilot test a data extraction form in Excel. We will extract information related to the type of collaborators and partners involved, the type of evidence synthesis, the training provided to collaborators and partners, the mode and frequency of their engagement, and the stage at which they were involved.

### Key informant interviews

We aim for broad relevance of this guidance for different health care areas. Therefore, we will seek input from external international individuals representing our 11 Ps on the proposed guidance via key informant interviews. We will utilize the results of the above systematic reviews, scoping review, evidence map, and methods study to develop draft candidate guidance items and seek perspectives on these as well as suggestions for additional items. We will develop a semi-structured interview guide and invite key informants using purposeful sampling for a maximum variation of our 11 P categories. This will promote participation from low- and middle-income countries (LMICs) and across gender, sex, and other intersecting identity factors. We will utilize the factors for identification and invitation of individual representatives previously published by members of our team [46]. Our advisory group and the MuSE Consortium will suggest participants and we will use snowball sampling for additional interview participants by asking each key informant to identify others. Sample size for the interviews will depend on the available evidence from the systematic reviews, scoping review, and evidence map. Our collaborator and partner co-leads will determine when we have completed enough interviews to clarify the evidence from the reviews. We anticipate conducting between 3 and 10 interviews per collaborator and partner category, based on our previous experience with the MuSE Guidelines project [17].

Two researchers will analyze transcribed interviews using a six-phase process of thematic analysis [47]. The process of qualitative data analysis will use the steps of (1) familiarisation with data; (2) generation of initial codes within each transcript; (3) search for themes; (4) review of themes; (5) define and name themes; and (6) reporting of themes [47]. We will also assess whether identified themes vary by gender or sex and other equity-relevant PROGRESS-Plus characteristics, where possible. We will submit this project to the Bruyère Research Ethics Board for approval. We will use transcription software for the audio recordings and we will use NVIVO software for the qualitative analysis (https://www.qsrinternational.com/).

### International survey

We will use the results of the above systematic reviews and key informant interviews to draft a list of candidate items for guidance on collaborator and partner engagement. We will survey international collaborators and partners using this preliminary list of guidance items to gather opinions about each draft item. We will disseminate the survey broadly though the members of the MuSE Consortium and their networks as well as through relevant organizations, such as Campbell, Cochrane, and JBI (formerly the Joanna Briggs Institute). We will aim for diversity in the identification of survey recipients by inviting responses from LMICs and by targeted outreach, using methods our group has used previously, such as sharing the survey invitation through listservs, inviting key individuals or organizations as recommended by the MuSE members, and social media [48].

We will ask respondents to indicate their agreement with each draft guidance item and we will invite open-ended responses, as well as suggestions for additional items. We will tabulate the frequency of agreement for each proposed item. We will code the open-ended responses into themes using the methods described above. This survey will be submitted to the Bruyère Research Ethics Board.

### Step 3: consensus activities

We will use the results of the systematic reviews, key informant interviews, and survey to develop and refine the draft guidance and finalise the guidance based on a consensus approach at an in-person (if possible) or virtual meeting. We will use nominal group technique to gain agreement [49], which allows participants to indicate their ideas or opinions privately and then present these in a ‘round robin’ format with other consensus participants [50]. This method allows all collaborators and partners to have an equal voice [51].

### Step 4: dissemination

The guidance finalised at the consensus meeting will be included in a series of guidance documents addressing equity-oriented guidance on methods for collaborator and partner engagement in evidence synthesis, evaluating engagement in evidence synthesis, and reporting engagement in evidence synthesis. The core management team, collaborator and partner co-leads, and advisory group will co-produce a dissemination plan and work collaboratively to improve adoption of our guidance by key organizations. We will utilize the international MuSE Consortium network to disseminate guidance documents and share these via social media, including Twitter and blogs, as well as through the multiple listservs maintained by the members of the MuSE Consortium.

## Discussion

This work is intended to produce guidance for engaging collaborators and partners in conducting evidence syntheses, transparently reporting this engagement, and evaluating engagement. The guidance for collaborator and partner engagement in evidence syntheses will be tested through an iterative, consensus building process, via in-person meetings, teleconferences, and email correspondence with collaborators and partners of our 11 categories. We will ensure synergy with our related guidance for engagement in guideline development. Developing guidance for collaborator and partner engagement, guidance for evaluating, and reporting engagement may assist with the uptake of these resources by relevant organizations.

## Supplementary Information

**Additional file 1.**

## Data Availability

Not applicable.

## Abbreviations

LMICs: Low- and middle-income countries
MuSE: Multi-Stakeholder Engagement
PROGRESS: Place of residence, Race/ethnicity/culture/language, Occupation, Gender and sex, Religion, Education, Socioeconomic Status, Social capital
