# Supplementary material for: Protocol for the development of guidance for collaborator and partner engagement in health care evidence syntheses
Source: Syst Rev. 2023 Aug 2;12:134. doi: 10.1186/s13643-023-02279-1 (PMC10394942; doi:10.1186/s13643-023-02279-1)
Supplement: Supplementary file 1 — Additional file 1. [file 13643_2023_2279_MOESM1_ESM.docx]

| Name | Country | P Category | Project Role |
| --- | --- | --- | --- |
| Alba Antequera | Spain | PI | Research Team |
| Ana Marusic | Croatia | Peer Review Editor | P Category Co-Lead |
| Alison Riddle | Canada | PI | Research Team |
| Angus Gunn | UK | Product Maker | P Category Co-Lead |
| Anneliese Synnot | Australia | PI | Research Team |
| Arnav Agarwal | Canada | PI | Research Team |
| Asma Ben Brahem | Tunisia | Policymaker | P Category Co-Lead |
| Marc Avey | Canada | Program Manager | P Category Co-Lead |
| Behrang Kianzad | Denmark | Payer/Purchaser of health services | P Category Co-Lead |
| Bev Shea | Canada | PI | Research Team |
| Christine Laine | USA | Peer Review Editor | P Category Co-Lead |
| Christopher McCutcheon | Canada | PI | Research Team |
| Comfort Ekanem | Nigeria | Providers | P Category Co-Lead |
| Danielle Pollock | Australia | PI | Research Team |
| Davina Ghersi | Australia | PI | Research Team |
| Diana Ingram | USA | Provider | Research Team |
| Emily Cahill | USA | Program Manager | Research Team |
| Elise Berliner | USA | PI | Research Team |
| Elizabeth Ghogomu | Canada | PI | Research Team |
| Elena Parmelli | Belgium | PI | Research Team |
| Eve Tomlinson | UK | PI | Research Team |
| Francesco Nonino | Italy | PI | Research Team |
| Gabriel Rada | Chile | PI | Research Team |
| Glen Hazlewood | Canada | PI | Research Team |
| Holger Schunemann | Canada | PI | Core Team |
| Ian Graham | Canada | PI | Research Team |
| Imad Bou Akl | Lebanon | Provider | P Category Co-Lead |
| Ina Kopp | Germany | PI | Research Team |
| Jane Cowl | UK | Public | Research Team |
| Janet Hatcher Roberts | Canada | PI | Research Team |
| Janet Jull | Canada | PI | Research Team |
| Jennifer Hilgart | UK | PI | Research Team |
| Jennifer Petkovic | Canada | PI | Core Team |
| Joanne Khabsa | Lebanon | PI | Core Team |
| Jordi Pardo Pardo | Canada | PI | Core Team |
| Karen Head | UK | PI | Research Team |
| Karine Toupin April | Canada | PI | Research Team |
| Kevin Pottie | Canada | PI | Core Team |
| Lara Maxwell | Canada | PI | Research Team |
| Laura Dormer | UK | Peer Review Editor | P Category Co-Lead |
| Lawrence Mbuagbaw | Canada | PI | P Category Co-Lead |
| Leonila Dans | Philippines | PI | Research Team |
| Ligia Teixeira | UK | Program Manager | P Category Co-Lead |
| Lorenzo Moja | Italy | Payer/Purchaser of health services | P Category Co-Lead |
| Lyubov Lytvyn | Canada | Canada | Core Team |
| Marisha Palm | USA | Public | P Category Co-Lead |
| Michael Saginur | Canada | Provider | P Category Co-Lead |
| Navin Sewak | UK | Product Maker | P Category Co-Lead |
| Neal Haddaway | Sweden | PI | Research Team |
| Nevilene Slingers | South Africa | Program Manager | P Category Co-Lead |
| Olivia Magwood | Canada | PI | Core Team |
| Omar Dewidar | Canada | PI | Research Team |
| Pauline Campbell | UK | PI | Research Team |
| Peter Tugwell | Canada | PI | Core Team |
| Alex Todhunter-Brown | UK | PI | Core Team |
| Regina Greer-Smith | USA | Public | P Category Co-Lead |
| Richard Morley | UK | Patient | P Category Co-Lead |
| Roses Parker | UK | PI | Research Team |
| Rosiane Simeon | Canada | PI | Research Team |
| Sophie Glatt | UK | Product Maker | P Category Co-Lead |
| Soumyadeep Bhaumik | India | Peer Review Editor | P Category Co-Lead |
| Stephanie Chang | USA | Peer Review Editor | Research Team |
| Tamara Kredo | South Africa | PI | Research Team |
| Tamara Lotfi | Canada | PI | Research Team |
| Tanja Kuchenmüller | Germany | Policymaker | P Category Co-Lead |
| Tanya Horsley | Canada | PI | Research Team |
| Thomas Concannon | USA | PI | Research Team |
| Thurayya Arayssi | Qatar | PI | P Category Co-Lead |
| Vivian Welch | Canada | PI | Core Team |
| Wojtek Wiercioch | Canada | PI | Research Team |
| Zachary Munn | Australia | PI | Research Team |
